# Morphology of the Epidermis of the Neotropical Catfish *Pimelodella lateristriga* (Lichtenstein, 1823) with Emphasis in Club Cells

**DOI:** 10.1371/journal.pone.0050255

**Published:** 2012-11-30

**Authors:** Eduardo Medeiros Damasceno, Juliana Castro Monteiro, Luiz Fernando Duboc, Heidi Dolder, Karina Mancini

**Affiliations:** Departamento de Ciências Agrárias e Biológicas, Centro Universitário Norte do Espírito Santo, Universidade Federal do Espírito Santo, São Mateus, Espírito Santo, Brasil; Emory University School of Medicine, United States of America

## Abstract

The epidermis of Ostariophysi fish is composed of 4 main cell types: epidermal cells (or filament containing cells), mucous cells, granular cells and club cells. The morphological analysis of the epidermis of the catfish *Pimelodella lateristriga* revealed the presence of only two types of cells: epidermal and club cells. The latter were evident in the middle layer of the epidermis, being the largest cells within the epithelium. Few organelles were located in the perinuclear region, while the rest of the cytoplasm was filled with a non-vesicular fibrillar substance. Club cells contained two irregular nuclei with evident nucleoli and high compacted peripheral chromatin. Histochemical analysis detected prevalence of protein within the cytoplasm other than carbohydrates, which were absent. These characteristics are similar to those described to most Ostariophysi studied so far. On the other hand, the epidermal cells differ from what is found in the literature. The present study described three distinct types, as follows: superficial, abundant and dense cells. Differences among them were restricted to their cytoplasm and nucleus morphology. Mucous cells were found in all Ostariophysi studied so far, although they were absent in *P. lateristriga*, along with granular cells, also typical of other catfish epidermis. The preset study corroborates the observations on club cells' morphology in Siluriformes specimens, and shows important differences in epidermis composition and cell structure of *P. lateristriga* regarding the literature data.

## Introduction

The animal epidermis is a tissue exposed on the body surface, which is in direct contact with the surrounding environment. It acts in numerous functions related to the interface organism/environment, being also involved in the protection mechanisms against physical, chemical and biological agents, such as pathogens. In specimens of the Superorder Ostariophysi, epidermis is composed of four cells types: epidermal, mucous, granular and club cells [1]–[8].

Epidermal cells, also known as filament containing cells, are the smallest and most numerous cells, being the major epithelium covering cells, and are found all over the epiderm, from basal to superficial layers [1], [3]. Mucous and granular cells are conspicuous round cells with peripheral flattened nucleus, located on the apical region of the epithelium [1]. They are important functional constituents of fish epidermis producing a glycoprotein that, when secreted, lubricates the skin and favors the animal's motion inside water.

Club cells are distributed throughout the epidermal layer and possess cytoplasm filled with material to be secreted and one centered nucleus [1]–[4], [7], [9]–[11]. They are found in different fish groups associated with distinct functions [12]. Zaccone and collaborators [13] demonstrated the presence of serotonin in these cells and suggested a pheromonal function. Other authors attributed an antipathogenic function to these cells [14]–[16], or suggested a phagocytic function [17]. Chondroitin and keratin were also found in some fish [12], suggesting a healing function, thus helping on repair of damaged tissue [18].

Among their various functions in most Ostariophysi, the club cells are related to production, storage and release of the alarm substance, leading to intra or interspecific alarm reaction in phylogenetically close species [16], [19]–[22]. In Ostariophysi, the alarm reaction is triggered when individuals are threatened or preyed upon his injured epidermis. This event causes disruption of the club cells cytoplasmic membrane, resulting in exposition and releasing of cytoplasmic content into the water, which is detected by other individuals in the school [16], [21], [23]–[26].

Despite several studies have already described the club cells in fishes, their results were based on cell-function relations, characterizing ecological and behavioral studies [10], [21], [27]–[33].

We choose the genus *Pimelodella* Eigenmann & Eigenmann 1888 due to the lack of morphological studies of the epidermis of Neotropical fishes. *Pimelodella* is one of the most diverse genera of the Heptapteridae family, with 82 described species until the conclusion of this work [34], [35]. They are distributed from southern South America to Panama and Central America [36]. The genus is popularly known as “mandi-chorão” (*crying mandi*) because of the sound it makes during his capture. *Rhamdia* and *Pimelodella* are among the most common Heptapteridae in South America, being endemic to Neotropical regions, however, its biology is poorly known [37].

Therefore, this study aimed to describe the structure of the skin of the catfish *Pimelodella lateristriga* (Lichtenstein, 1823) with emphasis on club cell morphology.

## Materials and Methods

The collections of *Pimelodella lateristriga* were performed in two points of São Mateus river basin (Espírito Santo state, Brazil) (PELD 1: 18°39′00.8″S and 40°05′39.9″W and PELD 2: 18°39′02.2″S and 40°07′23.4″W) during the months of August and September of 2011. All Brazilian rivers are considered public area; however, we were authorized directly by all owners to have access to the collection areas. Catches were made using a trawl net, under SISBIO license – permanent license to zoological material sampling – number 19158-1; Prof. Dr. Luiz Fernando Duboc.

For the identification, the species were fixed in the field with formalin 10%. This identification was made by Prof. Dr. Leonardo Ferreira da Silva Ingenito at the lowest taxonomic level. Surplus copies were listed in the Zoological Collection of the North Capixaba [CZNC - CEUNES/UFES: number CZNC 72 (PELD 1, 3 ex.) and number CZNC 65 (PELD 2, 10 ex.)].

For light and transmission electron microscopies, the species were anesthetized with Benzocaine 0,5 g/l before dissection. All skin fragments were taken from the sacrificed individuals.

### Methods

**Figure 1:**
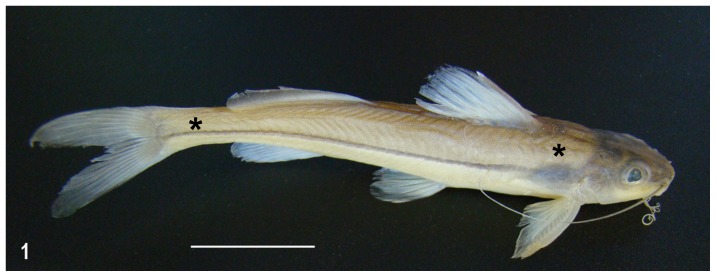
*Pimelodella lateristriga*. The asterisks indicate the cranial and caudal sections.

Light Microscopy: *P. lateristriga* skin fragments with approximately 1 cm^3^ were removed from the cranial and caudal regions of the animals (asterisks, Fig. 1) and fixed with Bouin's solution for 24 hours at 4°C. The fragments were washed, dehydrated in ascending ethanol series, clarified in xylene and then routinely embedded in paraffin (Paraplast). After embedding, the samples were sectioned (7 µm thick) and stained with Harris hematoxylin. For cytochemical procedures, the slides were stained with Mallory's trichrome, periodic acid-Schiff (PAS) and Bromophenol Blue. Mallory's trichrome method was employed to mark connective tissue areas. To do so, the slides were stained with Harris hematoxylin, then rinsed in 0.5% Acid Fuchsin aqueous solution and bathed in a solution of Aniline Blue 0.5%–2% Orange G - 1% phosphotungstic acid. In the glycoproteins detecting method (PAS) the slides were washed with 1% periodic acid, dipped in Schiff reactive, and counter-stained with Harris hematoxylin. In Bromophenol Blue technique, for proteins detection, slides were washed with 1% Bromophenol Blue aqueous solution and rinsed with 0.5% acetic acid.

Transmission Electron Microscopy: skin fragments with approximately 1 mm^3^, were removed from the cranial and caudal regions of the animals. Tissues were fixed by immersion in Karnovsky solution (2.5% glutaraldehyde, 4% paraformaldehyde in 0.1M sodium phosphate buffer, pH 7.2) for 24 hours at 4°C. The material was washed, post-fixed with 1% osmium tetroxide, dehydrated in increasing series of acetone, infiltrated, and embedded in epoxy resin. The embedded material was sectioned and collected on copper grids, thus contrasted with uranyl acetate and lead citrate solutions.

Scanning Electron Microscopy: skin fragments with approximately 1 cm^3^, were fixed by immersion in Karnovsky solution (glutaraldehyde 2.5%, 4% paraformaldehyde in 0.1 M sodium phosphate buffer, pH 7.2), washed and bathed in sucrose solutions (0.5M; 1M 1.5M, 2M, 3M, and 2.5M, in this order) for 24 hours in each solution at 4°C. The fragments were criofractured in liquid nitrogen, post-fixed in 1% osmium tetroxide and dehydrated in increasing series of ethanol. Thus, the samples were critical point dried and coated with gold/palladium. For ultrastructural characteristics, the micrographs were analyzed using measurement tools from the imaging software Adobe Photoshop.

Stereological and morphometric analysis: Micrographs were taken from different randomly chosen fields at a 400× magnification. The volume density (%) of skin components (epithelial cells, club cells, connective and muscle tissues) was obtained by the stereological methods described by [38]. Stereology was employed using a test-system with 165-test-points over a known area. The volume densities of the structures were estimated as *Vv[structure] = Pp[structure]/P_T_*, being Pp the number of points that were superimposed over the structure and P_T_ the total number of test-points contained in the area surrounded by the frame (grid). The connective tissue height, nuclear and cellular area (epithelial and club cells) were measured with a 400× magnification, using the software Image Pro-Plus. Fifteen micrographs of the anterior and posterior regions were used for measurements, being all cells from both regions measured. The means were compared with the Mann-Whitney “U” test with significance level of α = 0.05.

## Results

**Figure 2:**
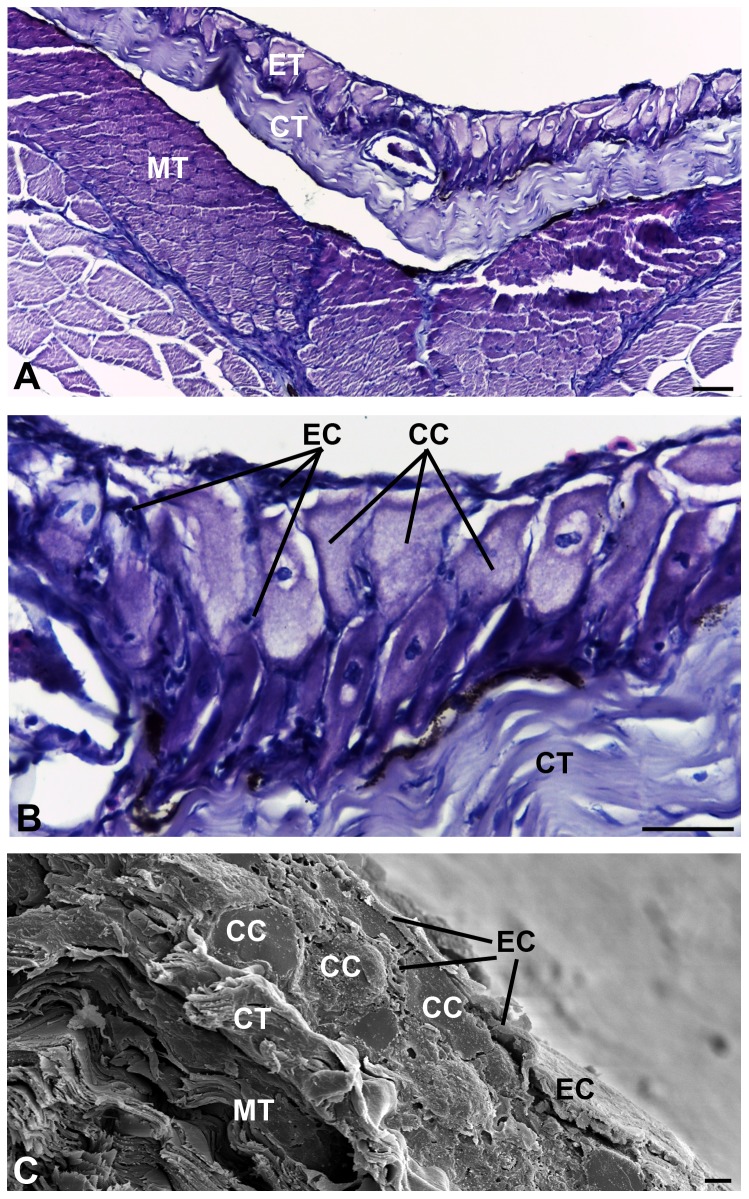
Tegument. (**A**) Light micrograph of the tegument showing an epithelial tissue (ET) supported by a connective tissue (CT) and a skeletal muscle tissue (MT). (**B**) Light micrograph of the stratified epithelial tissue composed by epidermal (EC) and club cells (CC). (**C**) Scanning electron micrograph showing the epithelial tissue with epidermal (EC) and club cells (CC), the connective tissue (CT) and the skeletal muscle tissue (MT). Scale bars: 25 µm (A); 15 µm (B) and 20 µm (C).

The skin of *Pimelodella lateristriga* is composed of a stratified epithelium, which is supported by a thick layer of dense irregular connective tissue (38.19%) and a wide muscle tissue (32.53%) (Figure 2A, C). The epithelium is composed of two morphologically distinct cell types: the epidermal cells (15.91%) and the club cells (13.31%) (Fig. 2B, C). The epidermal cells are small, with average area of 1,475.74 µm^2^, when comparing the conspicuous club cells that have average area of 2,991.68 µm^2^. These two cell types form a heterogeneous stratified epithelium composed of small flattened cells and large globular cells (Fig. 2B, C). The number of layers varies according to the disposal and heterogeneity of cell types, with generally two layers of club cells containing epidermal cells interspersed among them (Fig. 2B–C).

Epidermal cells are irregularly flat shaped, dense nucleus and discrete cytoplasm. They are well distributed across the epithelium, but preferably observed in surface regions defining its apical region (Fig. 2B–C). By transmission electron microscopy, it is possible to verify that the epidermal cells exhibit three distinct phenotypes, here called “superficial”, “abundant” and “dense”.

**Figure 3:**
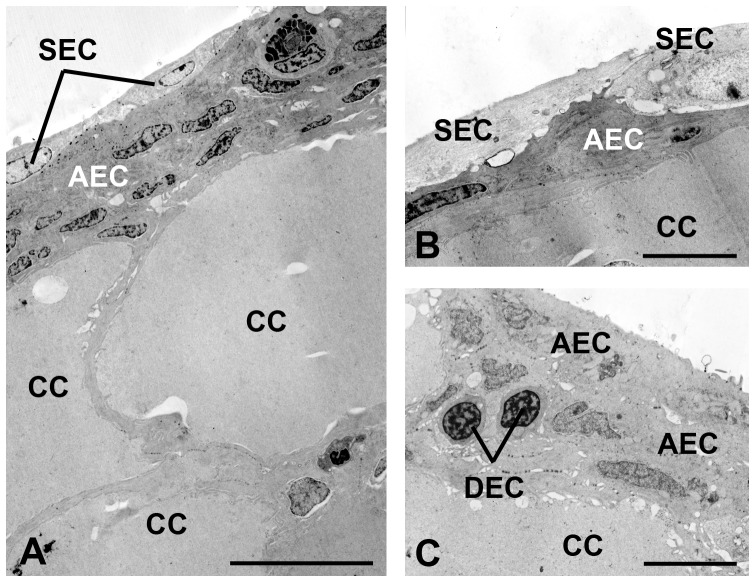
Transmission electron micrographs of epidermis with emphasis in the epidermal cells. The epidermis is composed by distinct cell types: club cell (CC), superficial epidermal (SEC), dense epidermal (DEC) and abundant epidermal (AEC). Scale bars: 10 µm (A) and 5 µm (B, C).

**Figure 4:**
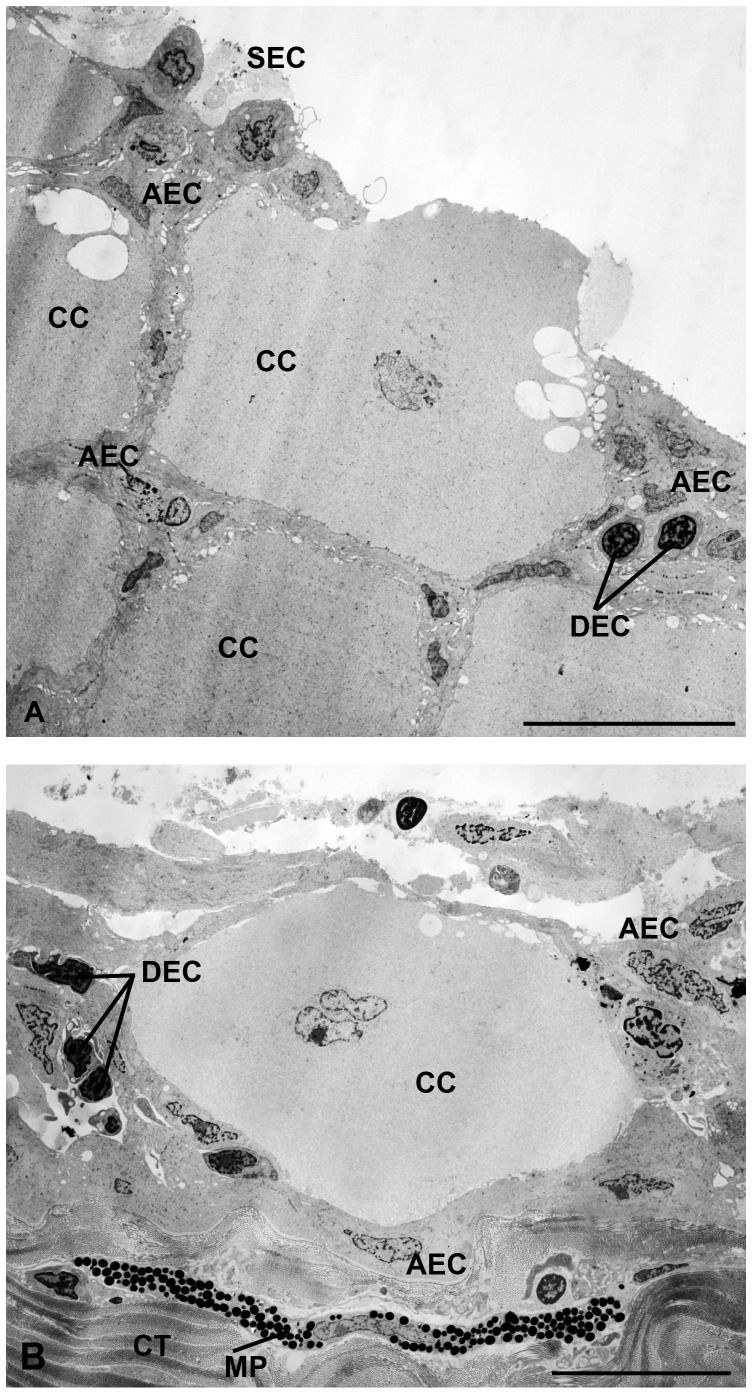
Transmission electron micrographs of epidermis with emphasis in the club cells. The epidermis is organized with conspicuous club cells (CC) and numerous small epidermal cell, as superficial epidermal (SEC), dense epidermal (DEC) and abundant epidermal (AEC) one. Note the connective tissue (CT) with melanophore (MP). Scale bars: 10 µm (A, B).

The “superficial” cells are flattened and located on the surface of the epithelium; show electron lucid cytoplasm and slightly condensed chromatin within the nucleus (Fig. 3A, B, 4A). The “abundant” cells are flattened, spread across the epithelium, showing dense cytoplasm and nucleus with condensed chromatin areas (Fig. 3A–C, 4A–B). The so called “dense” cells are round, distributed throughout the epithelium, but in small amounts, showing dense cytoplasm and globular nucleus with condensed chromatin regions (Fig. 3C, 4A–B)

Either light or electron microscopy assay did not show any mucous and/or granular cells.

**Figure 5:**
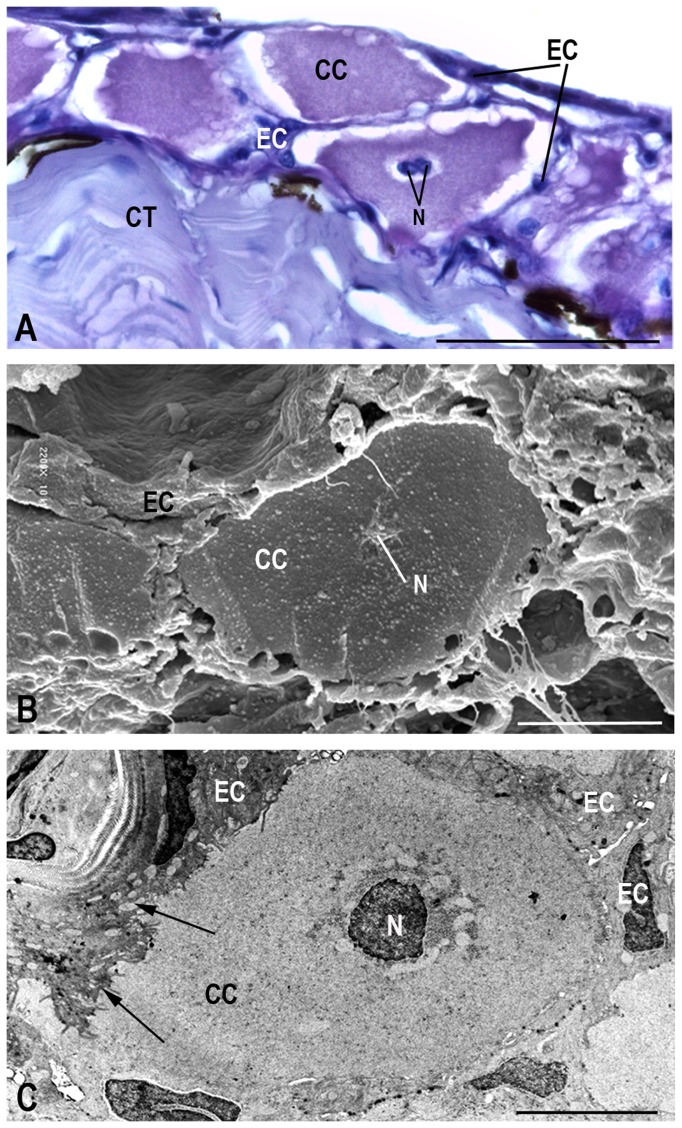
Light (A), transmission (B) and scanning (C) micrographs of club cells. The club cells (CC) possess a central nucleus (N) and are surrounded by epidermal cells (EC). In (**C**), presence of invaginations (arrows) of the club cell membrane. Connective tissue (CT). Scale bars: 24 µm (A); 10 µm (B) and 5 µm (C).

**Figure 6:**
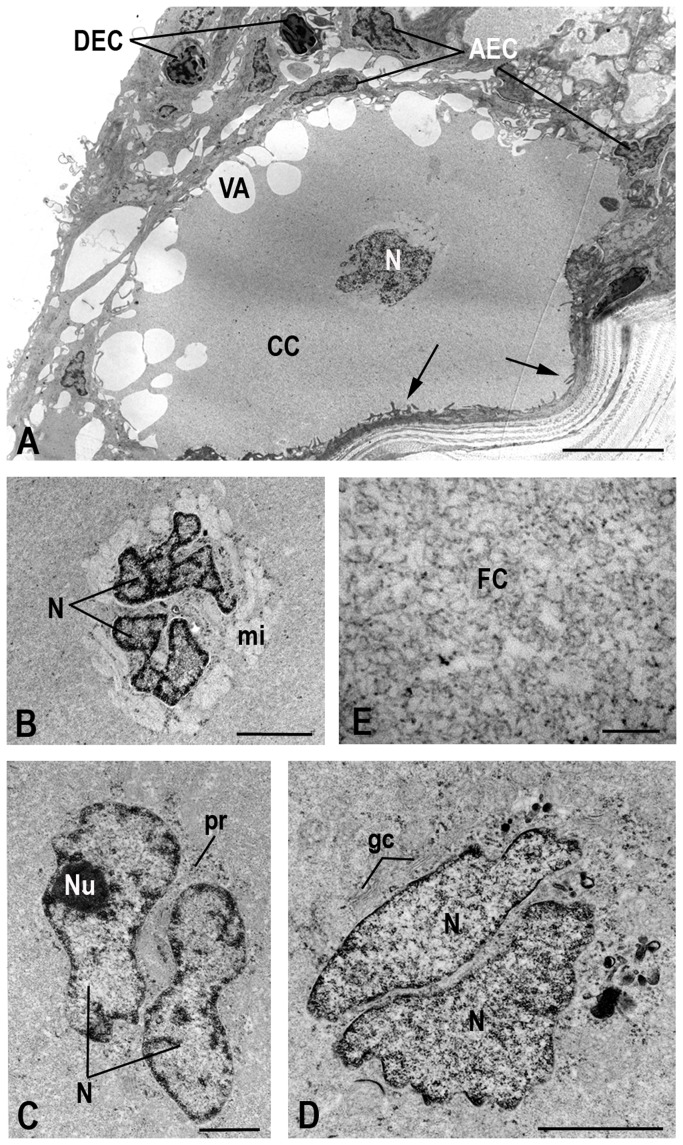
Transmission electron micrographs of club cells. (**A**) Club cell (CC) with central nucleus (N), membrane invaginations (arrows) and vacuoles (VA). Dense (DEC) and abundant (AEC) epidermal cells. (**B**) to (**D**) Perinuclear regions evidencing the presence of two nuclei in each club cell. The organelles are located next to the nuclei: mitochondria (mi), polirribossome (pr) and Golgi complex. Nucleoli (Nu). (**E**) Detail of the fibrillar cytoplasm (FC) with no vesicular secretion. Scale bars: 0.2 µm (E); 0.5 µm (A); 1 µm (C); 2 µm (B, D).

Club cells are arranged in two layers, constituting the largest extension of the epithelium. Skin fragments of cranial and caudal portions revealed no differences in the occurrence, density or morphology of club cells (Table 1). They are found mainly in the middle region, rarely reaching the apical surface (Fig. 2B–C). These cells show elongated and globular shapes (Fig. 2B–C, 4A–B, 5A–C, 6A). The nucleus is always central, measuring 1,325.73 µm^2^ in area (Fig. 5, 6). Two nuclei are found per cell, very close to one another, with irregular shape and slightly condensed chromatin, although with peripheral regions of compression and prominent nucleoli (Fig. 6A–C–E).

**Table 1:** Club cells in the *Pimelodella lateristriga* skin (n.s. = not significant).

Body Portions	Club cells					
	Occurrence	Localization	Volume density (%)	Shape	Cytoplasmarea (µm^2^)	Nucleus area (µm^2^)
**Cranial**	Present	middle region, rarely reaching the apical surface	14,99	Elongated/globular	2,038.48	1,371.18
**Caudal**	Present	middle region, rarely reaching the apical surface	11,63	Elongated/globular	1,293.47	1,280.23
**Mann-Whitney**	-	-	n.s. (n = 22, U = 31.5, p = 0.056)	-	n.s. (n = 69, U = 509.0, p = 0.476)	n.s. (n = 69, U = 125.5, p = 0.427)

The cytoplasm of club cells is rather poor in organelles and rich in non vesicle secretion (Fig. 6). The few observed organelles (endoplasmic reticulum, Golgi complexes, polyribosomes and mitochondria) are located in the perinuclear region (Fig. 6B–C), while the rest of the cytoplasm is filled with a filamentous substance (Figure 6F). Therefore, the cytoplasmic content can be separated into two regions: one light and electron lucid around the nucleus and other abundant and electron dense, which occupies nearly the entire cytoplasmic volume (Fig. 6B–C). Large vacuoles are occasionally displayed in the peripheral cytoplasm (Fig. 6A). The plasma membrane shows invaginations throughout its length, making the cell surface irregular and associated with the epidermal cells (Fig. 6A).

**Figure 7:**
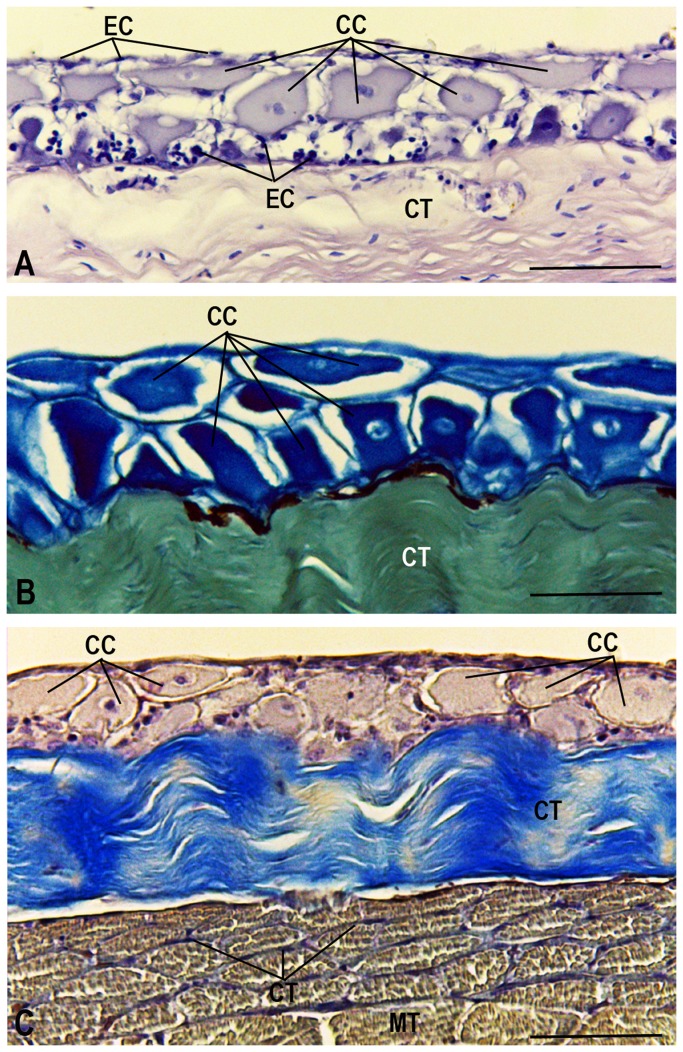
Cytochemistry. (**A**) PAS method; (**B**) Bromophenol Blue method and (**C**) Mallory trichrome method. Epidermal cells (EC), club cells (CC), connective tissue (CT) and skeletal muscle tissue (MT). Scale bars: 15 µm.

The club cells cytoplasm shows low glycoproteins content, as determined by the PAS technique (Fig. 7A), while the Bromophenol Blue technique, used for proteins detection, shows positive reaction (Fig. 7B).

Underneath the epithelium is a layer of loose connective tissue with fibroblasts and associated melanophores (Fig. 4B). Below the loose connective tissue, is a thick layer of dense connective tissue with an average thickness of 1,347.63 µm, highlighted in blue by Mallory's trichrome (Fig. 7C). Likewise, below dermis, lays a thick skeletal muscle tissue layer, as evidenced in red/brown by Mallory's trichrome (Fig. 7C).

## Discussion

In the present study, we analyzed skin fragments of two distinct body regions in *Pimelodella lateristriga*: cranial and caudal. However, the statistical analyzes revealed no differences regarding the volume density and area of club cells between those body parts (Table 1). Furthermore, no morphological differences were observed.

The epithelium of *P. lateristriga*, as in all Ostariophysi species studied so far, is classified as stratified and heterogeneous, showing different cell types. However, the number of layers and thickness of the epithelium is quite varied across species. In general, there is a thick stratified epithelium, as in Siluriformes [4], [7], [11], [39], in Characiformes [2] and in non-Ostariophysi such as Aguiliformes [40]. *P. lateristriga* epithelium is formed only by one or two layers of club cells lined with small epidermal cells. Club cells are concentrated in the middle region of the epithelium, thus not reaching the surface. This location corroborates previous findings, for both marine and fresh water Siluriformes [4], [7], [31], [41].

The presence of two different cell types within *P. lateristriga* epidermis - epidermal and club cells - is not shared by other Siluriformes, which had their epithelial structure described [2], [4], [9], [7], [10], [11], and Cypriniformes [5], [8], [10]. The main difference was the lack of mucous and granular cells in *P. lateristriga*, which are common and abundantly distributed within the epithelium of other species.

In general, mucous cells are characterized by their large size (similar to club cells), containing cytoplasmic vesicles filled with PAS positive secretion; they are located in the apical region of the epithelium and possess pores in their plasma membrane, from where the mucous secretion is released. The absence of mucous cells in *P. lateristriga* is unexpected and would be related to environmental or seasonal factors, since the collections occurred in a short amount of time and in the same season. [10], studying the effects of steroids on the skin of *Phoxinus phoxinus* (Linnaeus, 1758) (Cypriniformes), noted that there is a close relationship between steroid action and the amount of both mucous and club cells.

Granular and mucous cells are morphologically similar [1]. However, granular cells' cytoplasm is PAS negative and filled with electron dense granules. As the mucous cells, its absence within the epithelium may be related to intrinsic and extrinsic factors.

The epidermal cells found in *P. lateristriga* were called “abundant”, “superficial” and “dense”, since there are no citations in the literature so far, even for other species (there is only a single set called epidermal cells). The majority of studies involving ultrastructure of fish skin use high magnification micrographs, which make it difficult to identify these cell types [1], [9], [42]–[44].

Regarding the Ostariophysi, the most striking feature of club cells is their size, being easily identified by light microscopy. Although they are considered club cells, (i. e. in the shape of a club) in *P. lateristriga* they are irregularly shaped, ranging from globular to elongate, which is, indeed, a common morphological variation. In *Phoxinus phoxinus* (Cypriniformes) the same cell type is characterized as having a club shape (*Phoxinus laevis* as a species cited in [19]; in *Astyanax mexicanus* (De Filippi, 1853) (Characiformes) it is described as oval [44]; and in Siluriformes it varies from globular to elongated. In the ariid catfish *Plicofollis argyropleuron* (Valenciennes 1840) it is described as elongated (*Arius tenuispinis* species cited in [4], whereas in *Ariopsis felis* (Linnaeus 1766) is globular (species *Arius felis* as cited in [31]. In the bullhead *Coreobagrus brevicorpus* (Mori 1936) this cell type shape varies from globular to elongated (cited as *Pseudobagrus brevicorpus* in [7], being elongated in the African sharptooth catfishes *Clarias gariepinus* (Burchell 1822) [3] and *Clarias batrachus* (Linnaeus 1758) [45], and globular in *Ictalurus punctatus* (Rafinesque 1818) [41].

The morphology of club cells in *P. lateristriga* is quite similar to that described for other Siluriformes [9], [41], [4] and Cypriniformes [5], [8] due to its large size, central location, presence of two nuclei and negative reaction to the PAS method.

The nuclear morphology of club cells in *P. lateristriga* is in agreement with those found in non-Ostariophysi, as the lamprey species *Ichthyomyzon bdellum* (Jordan 1885) (cited as *Ichthyomyzon unicuspis* [42], in Cypriniformes [5], [8] and other Siluriformes [2], [4], [7], [9], [45], [46]. The nucleus is described as central, irregularly shaped, with peripheral condensed chromatin and prominent nucleoli. In addition, club cells' nuclei in Siluriformes are usually binucleated, which is a strong indicative of intense cell activity, although sometimes they appear as mononucleated cells. However, in Characiformes (e.g. *Astyanax mexicanus*) the nucleus is usually single [44].

The cytoplasm of club cells in *P. lateristriga* is filled with homogeneously dispersed fibrillar material, similar to that described in other Siluriformes [1], [9] and Cypriniformes [1], [43]. However, in non-Ostariophysi such as eels and lampreys instead of regular fibrillar material, the cytoplasm showed spiral filaments arranged in bundles and oriented in different planes [1], [42]. In the peripheral region of the cytoplasm, next to the plasma membrane, large vacuoles could be seen in *P. lateristriga*, as well as demonstrated for *Ictalurus punctatus* [9], for the arrid *Ariopsis felis* [31] and for the non-Ostariophysi, such as eels [47]. Given the above, it is suggested that the vacuoles are structures typically found in club cells and may be directly related to the mechanism of secretion release.

In the perinuclear region, different from the rest of the fibrillar cytoplasm, are the club cells organelles (endoplasmic reticulum, Golgi complex, free ribosomes in the form of polyribosomes aggregates, mitochondria and lysosomes). Such cytoplasmic composition and organization are observed in most Siluriformes and Cypriniformes studied so far [1], [9], [43], including *P. lateristriga*. [1] described an unusual structure present in the perinuclear region of club cells in *Corydoras aeneus* (Gill 1858) (Siluriformes): an aggregation of smooth surface vesicles with vesicles concentrically arranged around a central oval area. The central area was fibrillar and collapsed vesicles were occasionally found.

The cytoplasmic structure found in *Corydoras aeneus* was not observed in other Siluriformes as *P. lateristriga* and *Ictalurus punctatus* [9] or in Cypriniformes [1], [43]. This structure would be present only in certain Ostariophysi species, indicating a difference between club cells within the group.

The cytoplasm of club cells in *P. lateristriga* showed negative reaction to the PAS method, indicating absence of glycoproteins in its composition, as detected in other Siluriformes [9], [2], [11] and Cypriniformes [5], [8]. The cytoplasm of club cells showed positive reaction to the method of Bromophenol Blue, indicating high protein content, as observed in other Siluriformes [2], [9], [48]. The detection of protein and non-detection of glycoproteins supports the observation of a large amount of ribosomes/polyribosomes in *P. lateristriga* in comparison to the low occurrence of rough endoplasmic reticulum and Golgi complex.

The plasma membrane of club cells in *P. lateristriga* showed remarkable invaginations, unlike the observed by [1] in *Corydoras aeneus* (Siluriformes) and in *Carassius auratus* (Cypriniformes). Such invaginations confer cell adhesion, essential to the epithelium that is usually submitted to pressure and friction.

This study aimed to describe the morphology of the epidermis of a fish without focusing on functional aspects. There are few studies in the literature regarding to electron microscopy. Thus, the present findings represent a benchmark in the epidermal ultrastructure of a Neotropical species from the order Siluriformes. Furthermore, the existing studies that use high magnification micrographs do not allow the study of organization and composition of the epidermis. Therefore, further analyses are necessary, due to differences between morphological and cytochemical/immunocytochemical studies on the epidermis of the fish species studied so far, especially in electron microscopy in an attempt to a more precise characterization of the components of the epidermis in Ostariophysi.
